# Dual-acting compounds that deliver an acidifying domain via zanamivir to the virus surface are potent influenza inhibitors

**DOI:** 10.1126/sciadv.aei8633

**Published:** 2026-11-06

**Authors:** Charley Mackenzie-Kludas, Wen-Yang Wu, Betty Jin, Jennifer L. McKimm-Breschkin, Ee Ling Seah, Paul Jones, Emily Fairmaid, Celeste M. K. Tai, Ding Yuan Oh, Peter J. Jenkins, Aeron C. Hurt, Lorena E. Brown

**Affiliations:** ^1^ The Department of Microbiology and Immunology, The University of Melbourne at the Peter Doherty Institute for Infection and Immunity, Melbourne, Australia.; ^2^ AusBio Ltd., Melbourne, Australia.; ^3^ The World Health Organisation Collaborating Centre for Reference and Research on Influenza at the Peter Doherty Institute for Infection and Immunity, Melbourne, Australia.; ^4^ International Collaboration Unit, International Institute for Zoonosis Control, and the Institute for Vaccine Research and Development, Hokkaido University, Sapporo, Japan.

## Abstract

While contemporary antiviral development primarily strives to produce “lock and key” inhibitors, we present here a different approach to drug design that uses compounds devised to alter the microenvironment of the virion surface, making it incompatible with virus entry. We illustrate this strategy with inhibitors of influenza virus and show that compounds designed to produce a proton-rich mantle above the virion surface affect the ability of the viral hemagglutinin to interact with cellular receptors and gain entry to the cell. The compounds show exceptional antiviral activity both in vitro and in vivo and protect against influenza illness in ferrets and in mice after a single dose, either therapeutically or prophylactically.

## INTRODUCTION

Currently licensed antiviral drugs for influenza target the neuraminidase (NA) to prevent efficient release of virus from cells (*1*) or inhibit the functions of the viral polymerase (*2*). These drugs block steps in the replication cycle rather than preventing initial infection. Compounds that theoretically could stop viral entry by blocking the receptor-binding pocket on the cell-attachment glycoprotein, hemagglutinin (HA), have remained elusive, potentially due to the shallow nature of the pocket (*3*) and variability of surrounding amino acids. During infection, binding of the HA to its receptor on respiratory epithelial cells triggers endocytosis of the virus. Within the endosome, an increase in acidification causes a low-pH–induced conformational change to take place in the HA (*4*) to allow exposure of a hydrophobic “fusion peptide” and the extension and insertion of a coiled-coil trimer, tipped with the hydrophobic domain, into the endosomal membrane (*5*, *6*). This process culminates in fusion of the viral envelope with the endosomal membrane and the creation of a fusion pore through which the viral genome can escape for replication. The native form of the HA is metastable (*5*, *7*), and the shift to the low energy fusogenic form is irreversible (*8*). The low-pH conformation of the HA can no longer engage with cellular receptors (*9*).

We have taken the approach of synthesizing compounds that have a docking domain for anchoring to the viral surface and an acidic effector domain to create a local drop in pH to mimic the low-pH environment found inside the endosome, thus triggering premature initiation of the HA into a form unable to bind to and enter cells (Fig. 1A). Of the various compounds that we have produced that have antiviral function, here, we focus on two of these for more detailed investigation. The compound MD185 (Fig. 1B) provides an acidic environment via four aminomethanesulfonic acid groups connected in pairs to a pyromellitic acid backbone by d-aspartic acid, whereas the effector domain of MD345 (Fig. 1C) is derived from a single chain of seven cysteic acid repeats connected to a tricarboxylic acid backbone (see the Supplementary Materials). In both cases, the acidic domains are linked to a sialic acid derivative related to the NA inhibitor (NAI), zanamivir (*10*). Zanamivir binds to and inhibits the activity of the second virion surface glycoprotein NA, the functions of which include mediating efficient release of nascent virus from infected cells by removing sialic acid from virus receptors in the region of virus budding (*1*). We have chosen a zanamivir-like compound as a virus-docking device because of its high barrier to the emergence of resistance (*11*) and use the dimeric form which has been shown to have prolonged effectiveness in vivo compared with monomeric zanamivir (*12*). Here, we present data showing the potent in vitro and in vivo antiviral activity of these novel bifunctional anti-influenza compounds as well as some insight into their mechanism of action.

**Fig. 1.**
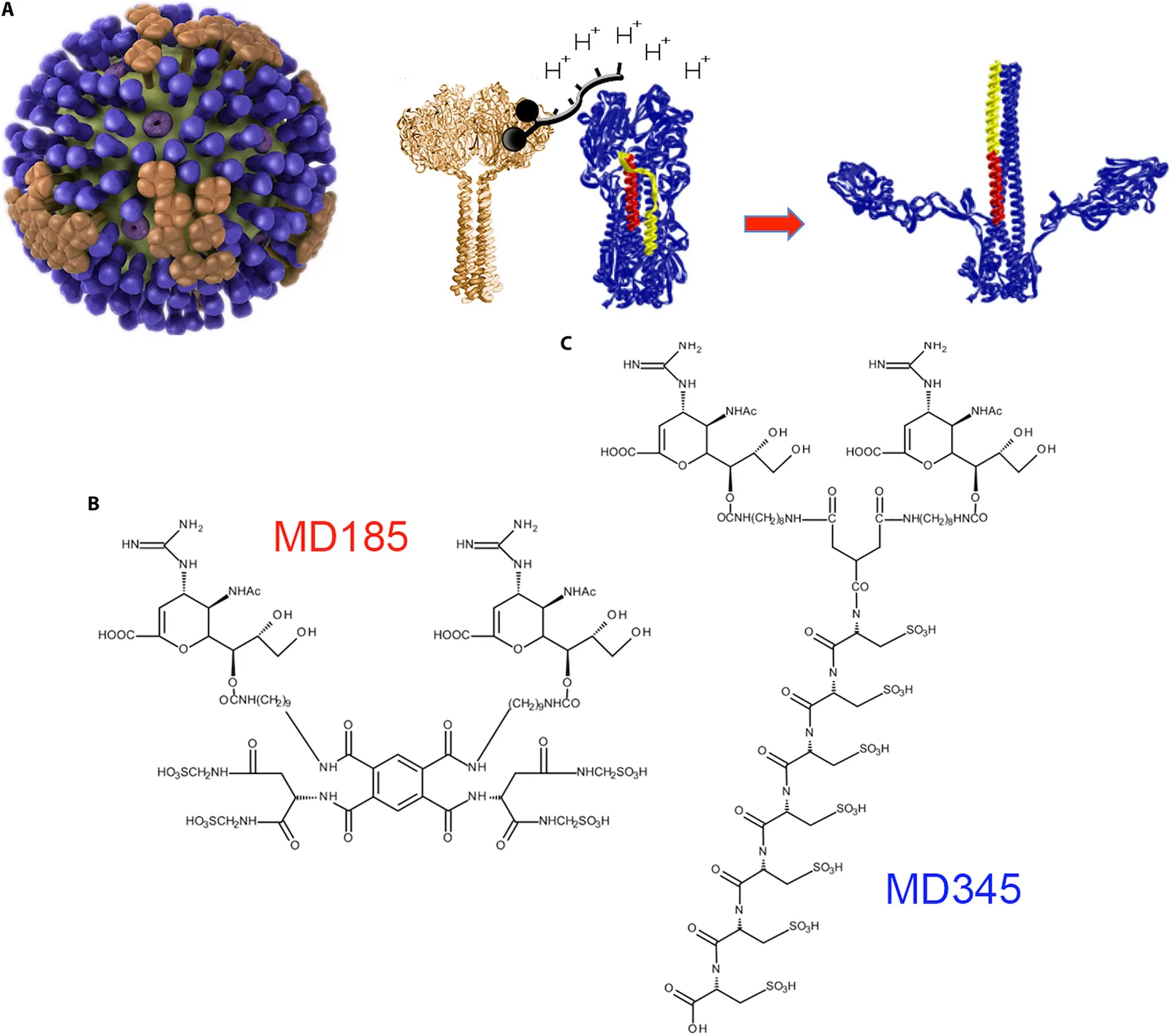
Structure and proposed action of compounds. (**A**) Graphical representation of the proposed mechanism of action. The modular design of the compounds includes an NA-binding anchor, a structural backbone, and an acidic effector domain. The compounds target the virion surface (left) and anchor to NA surface glycoprotein (brown). The acidic effector domain induces a drop in local pH (middle) that affects the neighboring HA molecules (blue) causing the irreversible change in the HA from the metastable to the low-pH fusogenic form (right), which precludes virion attachment to cell receptors. The illustration incorporates images of influenza virus from the Centers for Disease Control and Prevention, Atlanta, GA, USA; NA head domain, Protein Data Bank 4GZX, and the HA neutral form 1HGF. The extended intermediate fusion form is a schematic. (**B**) For MD185, the acidic domain consists of four aminomethanesulfonic acid groups connected to pyromellitic acid. (**C**) MD345 has a chain of seven cysteic acid repeats connected to a tricarboxcylic acid group. Both compounds are anchored to the virus by a pair of zanamivir homologs connected to the backbone via carbon chains. The molecular weight is 1852 and 2201 for MD185 and MD345, respectively, which are prepared as HCl salts (MWs, 2070.76 and 2419.76).

## RESULTS

### In vitro inhibition of influenza replication

When screened in cytopathic effect (CPE) reduction assays, both MD185 and MD345 were effective against a range of influenza strains (table S1). There was no toxicity at the highest dose of both drugs tested in Madin-Darby canine kidney (MDCK) cells, 100 μM for 72 hours, giving a selectivity index of >1000. For more precise evaluation, we used the plaque reduction assay (PRA). For testing NAIs, this assay normally requires incorporation of drug into the cell overlay medium, and this results primarily in reduction in plaque size due to inhibition of virus spread. However, as we wished to determine whether we could also demonstrate an impact on HA binding to cells, we pretreated viruses with the inhibitors, as in an antibody neutralization assay, and quantified the numbers of plaques. MD185 and MD345 showed 400- to 7000-fold greater activity than zanamivir against human and mouse-adapted H1N1 viruses, a recent H3N2 strain, and an influenza B virus (Fig. 2A).

**Fig. 2.**
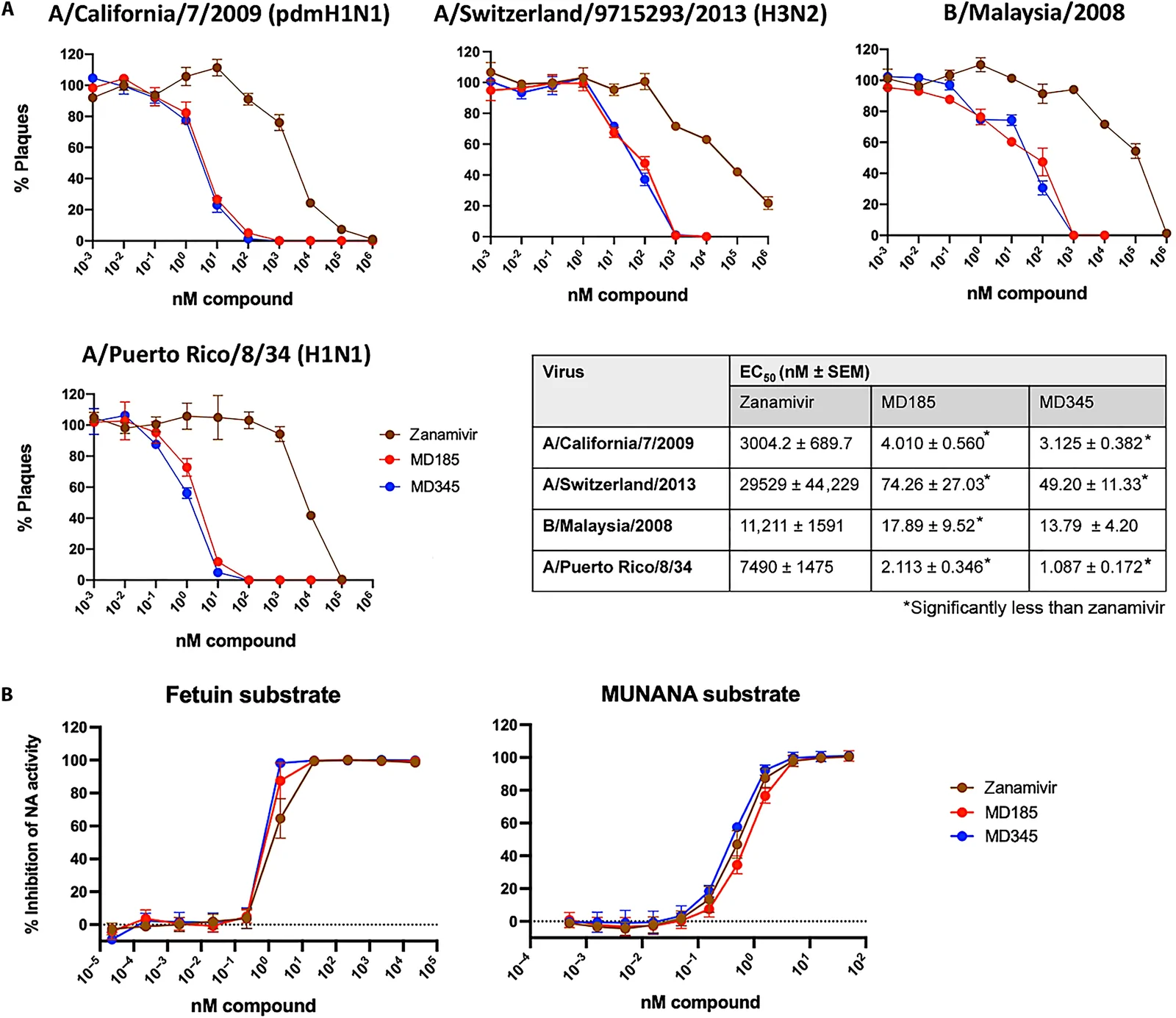
In vitro activity of compounds. (**A**) PRAs of A/California/7/2009 (100 PFU), A/Switzerland/9715293/2013 (100 PFU), B/Malaysia/2008 (100 PFU), and PR8 (120 PFU) influenza viruses by MD185 (red), MD345 (blue), or zanamivir (brown). All compounds were preincubated for 1 hour with virus before addition to MDCK cells and assayed for plaque formation. Data are expressed as a percent of plaques obtained with virus infection in the absence of compound. The table insert shows the median effective concentration (EC_50_) of the compounds against each virus with the SE of three determinations. For each virus, the EC_50_ values for MD185 and MD345 were significantly less than for zanamivir [*P* < 0.0001, one-way analysis of variance (ANOVA)]. (**B**) PR8 NAI activity of the compounds. Data are calculated as a percentage of NA activity relative to PR8 in the absence of inhibitor and represents the average of three independent assays, each performed in duplicate, using fetuin (left) or MUNANA (right) as substrate. The compounds showed no significant difference to zanamivir at any dilution in either assay (two-way ANOVA with Tukey’s multiple comparisons test).

Although this marked decrease in plaque number (fig. S1) is not expected to result from pretreatment of virus with an NAI, it was necessary to definitively establish whether we had just created a superior NAI due to additional steric hindrance of the NA active site by the acidic groups on the bivalent docking domain. To investigate this, we performed enzyme inhibition assays using both the large sialylated substrate fetuin and the small artificial substrate 2′-(4-methylumbelliferyl)-α-d-*N*-acetylneuraminic acid (MUNANA). As can be seen in assays using either substrate (Fig. 2B), there was no large improvement in the inhibition of NA activity by MD185 and MD345 compared with zanamivir. This demonstrates that, while the docking domains of the compounds do inhibit NA enzymatic function, this activity does not explain the very large increase in potency over zanamivir observed in the PRA, suggesting that the enhanced efficacy of the compounds is due to an additional mechanism.

From these data, it could be predicted that the compounds modulate infection by preventing both entry, through inactivation of the cell binding function of HA by the effector domain, and exit, via the enzyme inhibition of the NA anchor domain. To confirm this, we performed time-of-addition studies in MDCK cells infected with the A/Puerto Rico/8/34 (PR8) laboratory strain over a single cycle of replication (Fig. 3). MD185 or zanamivir was added to the cells at discrete intervals and then removed by thorough washing. In contrast to zanamivir, which had an inhibitory effect only when added at the time of viral exit from the cells, we observed a two-log reduction in viral titer when MD185 was present together with the virus during the adsorption phase, implying inhibition of viral attachment and entry. The anchor domain of the compound appeared to function analogously to zanamivir in its inhibition of virus egress.

**Fig. 3.**
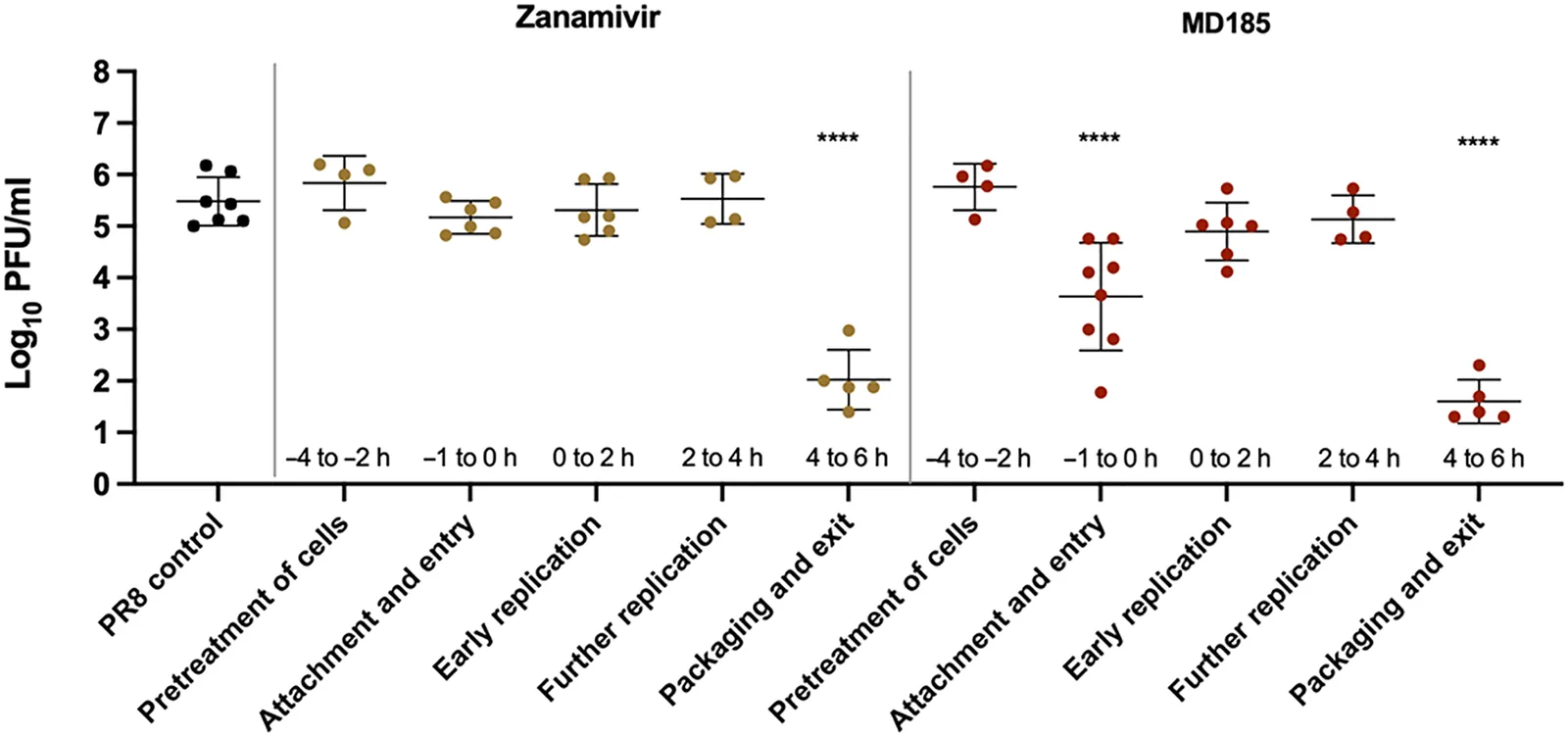
Time of addition assay shows inhibition of viral entry. MDCK cell monolayers in a 96-well plate were infected with PR8 virus at an MOI of 0.1. In PR8 control wells, the supernatants were harvested at 12 hours postinfection (hpi), and the amount of infectious virus was determined by plaque assay. To establish where in the virus replication cycle the compounds were acting, 5 μg of either MD185 (2.07 nmol) or zanamivir (15.05 nmol) was added to additional wells at different intervals either before or after infection with PR8 virus (*t* = 0 hour) as indicated above the *x* axis to capture the different stages of replication. Pretreatment of cells is in the absence of virus. For the attachment and entry stage, the virus was preincubated with zanamivir or MD185 (from −2 to −1 hour) and then allowed to adsorb to the cell monolayers from −1 to 0 hours. At the end of each treatment interval, the cells were thoroughly washed, medium was replaced and the infectious process allowed to continue to *t* = 12 hours when supernatants were again harvested, and infectious virus yields were determined by plaque assay. Data represent combined results from five individual experiments. ****, significantly different (*P* < 0.0001) to the PR8 control, one-way ANOVA. h, hours.

### Effect on HA conformation and function

To probe the effects of the compounds on HA, we first tested whether the neutral-pH–binding conformation had been disrupted after exposure to MD185 and MD345. Monoclonal antibodies (mAbs) specific for either the low (mAb 88/2)–, neutral (mAb 241)–, or both low and neutral (mAb 244)–pH conformations of A/Memphis/1/71 (H3N2) HA (*13*, *14*) were tested for their ability to bind to this virus after exposing it to the compounds. As conformational integrity is crucial for this test, we used a slot blot assay in which intact virus was treated with compound dissolved in pH 7.2 saline or control solutions and then serially diluted and bound to a nitrocellulose membrane. The membranes were then probed with the mAbs, and the relative intensities of the bands after development were determined. Treatment with either MD345 (Fig. 4, A and B, left) or MD185 (Fig. 4B, right) resulted in the loss of binding of the neutral-pH conformation–specific mAb 241 with a concomitant gain in binding of the low-pH conformation–specific 88/2 antibody, whereas the binding of the pH-independent mAb 244 remained unaffected (Fig. 4, A and B). The fact that pretreatment of the virus with the compounds produced a binding profile mimicking pretreatment with pH 4.9 citrate buffer is consistent with the compounds disrupting the neutral-pH conformation. It should be noted that the pH of the solutions of compounds, which was pH 7.0 after dilution in pH 7.2 saline (or pH 5.5 in unbuffered saline), was not sufficiently low to mediate the effects that we have observed, supporting the concept of creation of an acidic microenvironment at the virion surface through the binding of the compounds.

**Fig. 4.**
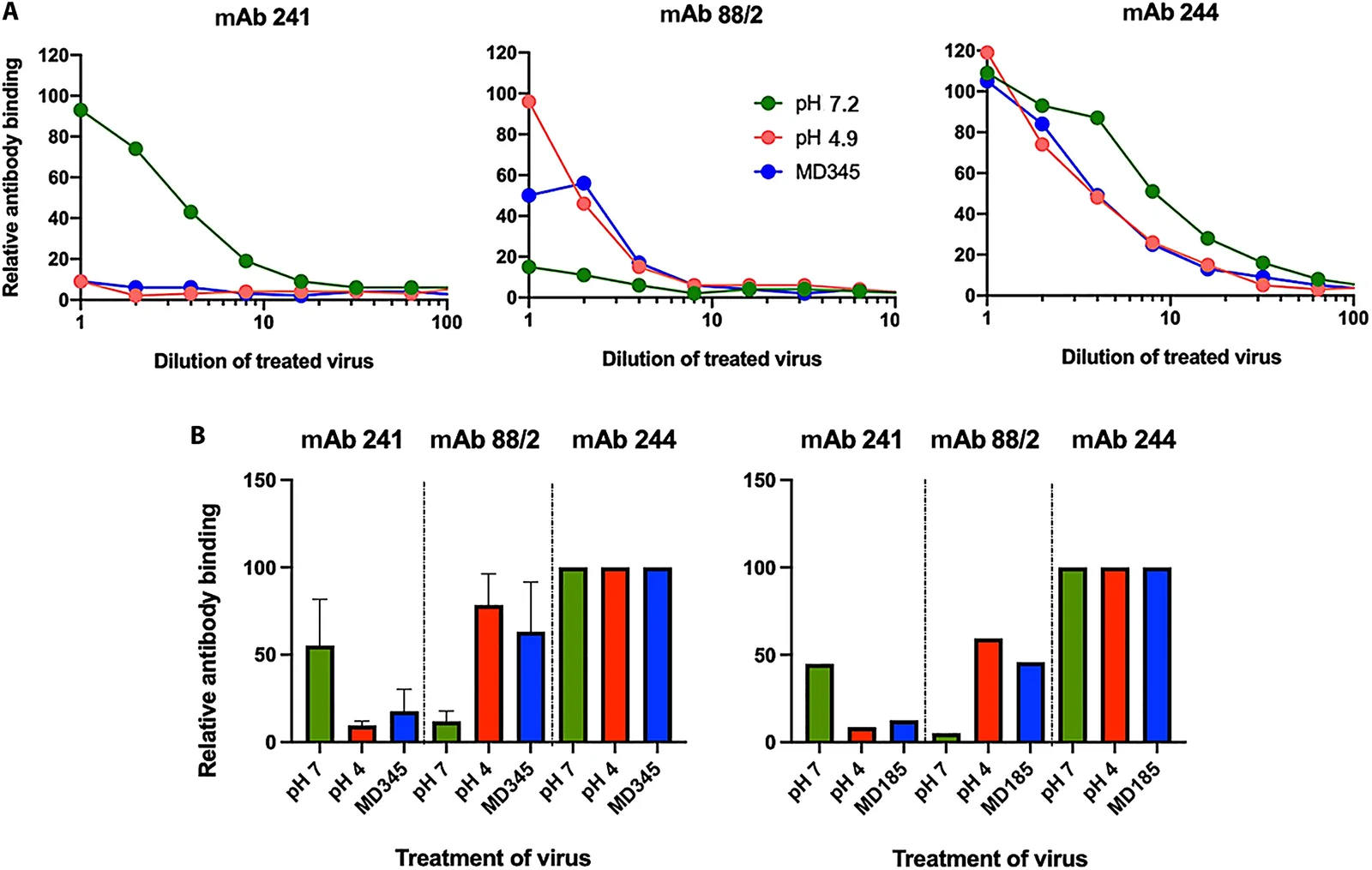
The compounds mediate disruption of the neutral-pH conformation of HA. (**A**) Influenza virus A/Memphis/1/71-Bel42 virus was treated with MD345 (blue), pH 4.9 citrate buffer (red), or pH 7.2 saline (green) and tested in a slot blot assay for binding to a panel of HA-specific mAbs. After serial dilution, the treated virus was bound to nitrocellulose and probed with: mAb 241, specific to the neutral-pH conformation of HA (left); mAb 88/2, specific for the low-pH–induced conformation (middle); or mAb 244, which can bind to either conformation (right). Data show a representative experiment. (**B**) Compiled replicates (left) of the initial dilution of the experiment shown in (A) from three independent experiments, in which the virus was treated with a large excess of MD345 or the pH 4.9 or pH 7.2 solutions for 2 hours before blotting and staining. The staining levels for each treatment are standardized to the staining obtained with mAb 244. Data for MD345 treatment were not significantly different to that of the low-pH treatment; one-way ANOVA. The pattern of reactivity is analogous to that observed in an additional experiment using MD185 (right). Results are expressed as the relative binding of the mAbs.

As a surrogate of how inducing the low-pH conformation in the HA of the virus affects its ability to interact with receptors, the compounds’ capacities to inhibit hemagglutination of chicken red blood cells were examined (fig. S2). In contrast to zanamivir, which failed to inhibit hemagglutination, both compounds showed marked inhibition, with MD345 being much more active.

### Efficacy against drug resistant NA mutants

When evaluating any new influenza antiviral compound, it is critical to determine its efficacy against known resistant viruses and additionally to determine whether fit, resistant mutants are readily selected after prolonged exposure to the inhibitor. Because the anchor domain is a zanamivir-like molecule, we tested efficacy against the A/NWS/G70C parent virus and its E119G zanamivir-resistant mutant (Fig. 5A) (*15*). In the PRA, both compounds were >200-fold more potent than zanamivir against the parent virus, and, while the E119G substitution reduced sensitivity to zanamivir by ∼500-fold, there was only a 12-fold reduction in sensitivity to MD185 and 33-fold reduction to MD345. Notably, the activity of the compounds against the mutant with decreased sensitivity to zanamivir was still more potent than was zanamivir against the wild-type (WT) virus.

**Fig. 5.**
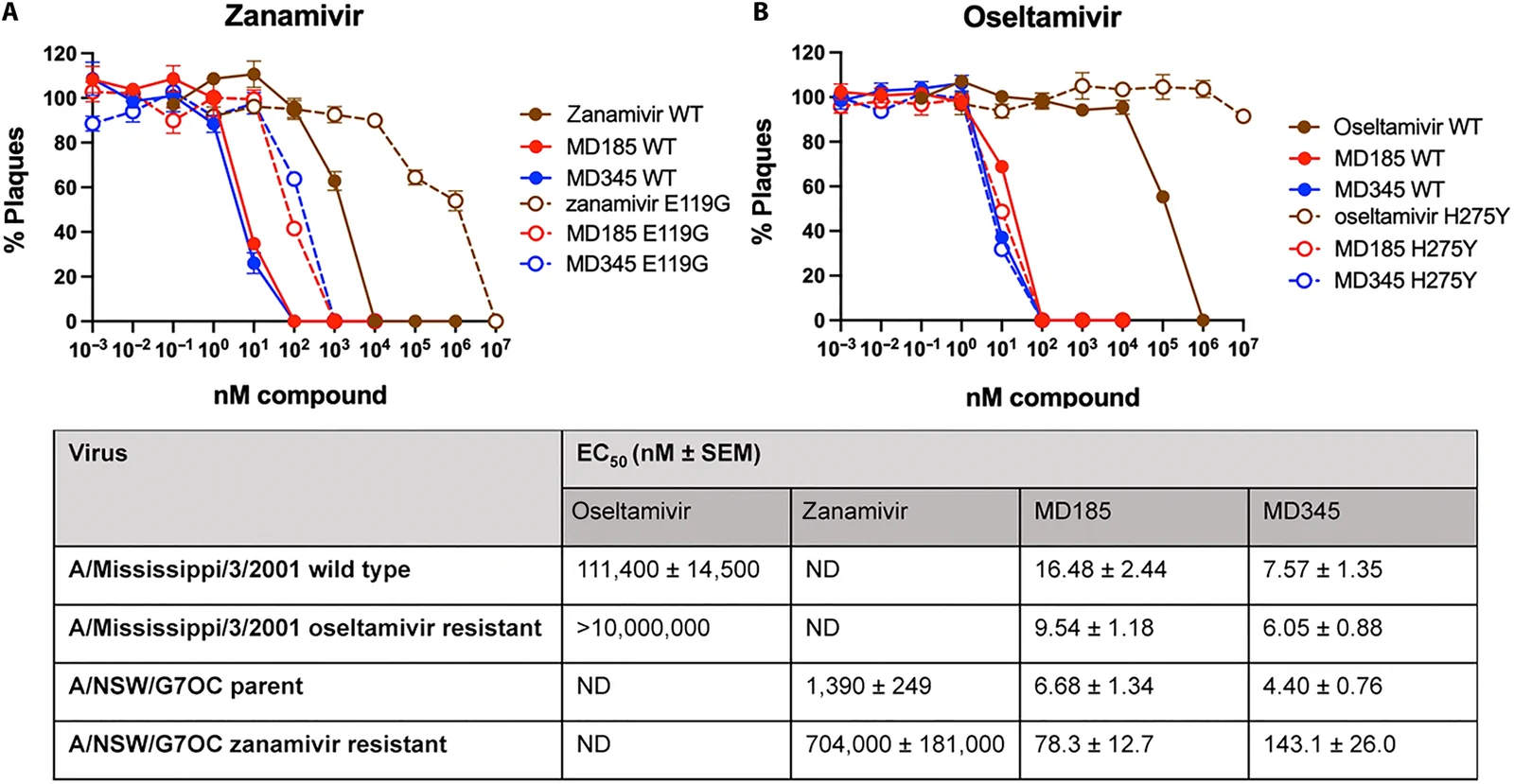
Activity of compounds against viruses resistant to licensed NAIs. (**A**) PRA of A/NWS/G70C H1N9 parent virus (solid lines) and its zanamivir-resistant mutant NA E119G (dashed lines) by MD185 (red), MD345 (blue), or zanamivir (brown). (**B**) PRA of A/Mississippi/3/2001 WT (solid lines) and its oseltamivir-resistant mutant NA H275Y (dashed lines) by MD185 (red), MD345 (blue), or oseltamivir (brown). The table insert shows the EC_50_ of the compounds against each virus with the SE of three replicate determinations. For each virus, with the exception of the oseltamivir-resistant virus that could not be calculated, the EC_50_ values for MD185 and MD345 were significantly less than for zanamivir (*P* < 0.0001, one-way ANOVA). MD185, but not MD345, showed slightly but significantly more activity against the WT compared with the oseltamivir-resistant virus (*P* = 0.04, unpaired *t* test) and both MD185 and MD345 showed significantly greater EC_50_ values against the parent versus zanamivir-resistant variant of A/NWS/G70C (*P* = 0.0007 and 0.0003, respectively).

The compounds were also tested for inhibition of the H1N1 A/Mississippi/3/2001 WT virus and the corresponding virus with an H275Y mutation in the NA that rendered it resistant to a commonly used NA-inhibitor, oseltamivir (Fig. 5B) (*16*). Against the WT virus, MD185 was 7000-fold and MD345 was 15,000-fold more potent than oseltamivir, and, while the H275Y mutation conferred >90-fold resistance to oseltamivir as predicted, neither MD185 nor MD345 showed significant differences in inhibition of the mutant and WT viruses.

### Selection of viruses with decreased drug sensitivity

To try to rapidly select for resistant variants, A/Perth/265/09 (pdmH1N1) and A/Fukui/45/04 (H3N2) viruses were passaged at a high multiplicity in the presence of 10 ng/ml (EC_90_) of MD185. After four passages in the compound, no H3N2 resistant variants were isolated. However, mixed plaque phenotypes were seen in the MD185-passaged pdmH1N1 virus (fig. S3), and two small plaques were picked and amplified for characterization. One of these was found to possess a Q136K NA substitution, a mutation shown previously to be selected in MDCK cell culture in the absence of zanamivir pressure (*17*), although no such change was observed in viruses passaged in the no drug/control arm of this experiment. The second had an E119K NA substitution accompanied by a G172E substitution in the HA. In a PRA (Fig. 6A), MD185 was around 80-fold more potent than zanamivir against the WT virus. On average, the Q136K and E119K mutations reduced inhibition by zanamivir by 50-fold and, at least, 4000-fold, respectively, but only by 10- and 80-fold with MD185. Although sensitivity to MD185 was somewhat reduced, it was still about 400- and 100-fold more effective than zanamivir against Q136K and E119K mutants, respectively. Thus, even with viruses containing substitutions conferring reduced NAI sensitivity, the acidic effector domain of the compounds provided robust antiviral activity.

**Fig. 6.**
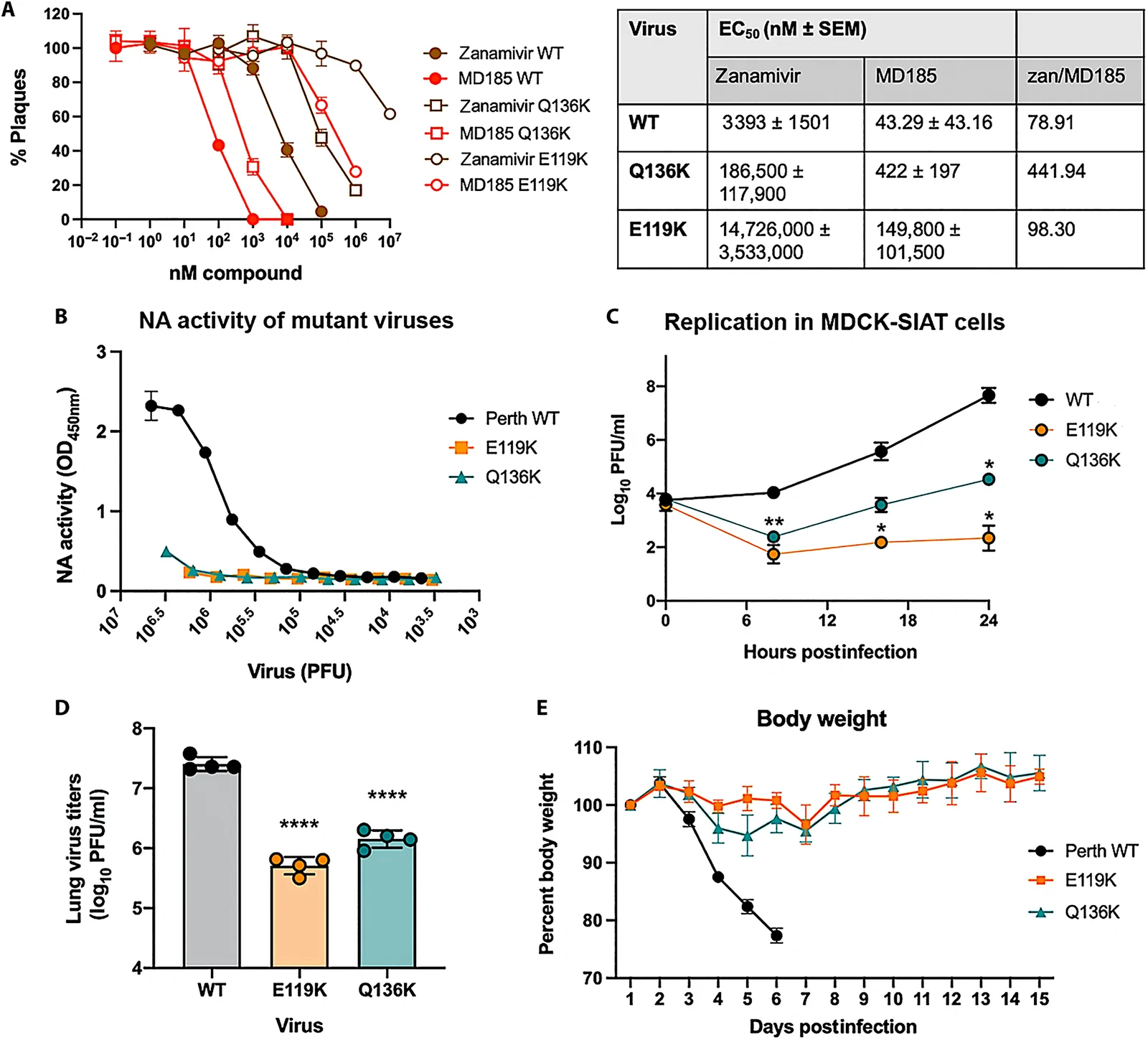
Activity of compounds against mutants with reduced susceptibility to MD185. (**A**) PRA of A/Perth/265/09 WT and its mutants NA E119K and Q136K by MD185 (red) and zanamivir (brown). Data represent means and SEM of triplicate samples of a representative of three individual experiments. The table insert shows the means of the EC_50_ ± SEM for the three experiments and the fold difference between the EC_50_ values for zanamivir and MD185. MD185 was significantly less than zanamivir for each virus comparison, *P* = 0.0021, 0.0071, and 0.0025 for WT, E119K, and Q136K, respectively (unpaired *t* test). (**B**) The NA enzyme activity of A/Perth/265/09 WT (black), mutant E119K (orange), and mutant Q136K (teal) was assessed using fetuin as a substrate. Data show a representative of three independent experiments performed in duplicate. (**C**) MDCK-SIAT1 cells were infected with 3000 PFU WT A/Perth/265/09 virus (black), mutant E119K (orange), or mutant Q136K (teal). Supernatants were sampled at 8, 16, and 24 hours and assayed by plaque formation in MDCK cells. The means and SEM of combined data from two individual replicate experiments each performed in duplicate are shown. Significantly different (*P < 0.05; **P < 0.01) from WT virus, two-way ANOVA with Tukey’s multiple comparisons test. (**D**) Mice (*n* = 4) were infected with 500 PFU of WT A/Perth/265/09 virus (black), mutant E119K (orange), or mutant Q136K (teal), and the lungs were sampled 3 days later, homogenized, and assayed for infectious virus by plaque formation in MDCK cells. Significantly different (****P < 0.0001) from WT virus, one-way ANOVA. (**E**) A second set of mice was infected as in (D), and weight loss and clinical signs were monitored at least once daily. All mice infected with the WT virus rapidly lost weight and were euthanized on day 6 at the humane endpoint. Shown are the means and SEM (*n* = 4).

Further characterization demonstrated that the NA activity of the mutants was barely detectable (Fig. 6B), suggesting that the normal functions of NA in cleaving sialic acid from the receptors on infected cells to promote efficient virus exit from cells would be compromised, reducing replication (*18*). To test this, we infected MDCK-SIAT 1 cells, which overexpress sialyl-alpha2,6-galactose moieties on the cell surface, and assessed virus yields over a 24-hour period (Fig. 6C). Both mutants showed decreased production of virus compared to the A/Perth/265/09 WT virus when replicating in the presence of a high density of cell surface receptor. At 24 hours postinfection (hpi), ∼3- and 5-log_10_ less virus was detected for the Q136K and E119K mutants, respectively. These data suggest that replication may also be compromised in vivo as seen with other mutants with low NA activity (*19*). This was confirmed by the reduced pulmonary virus titers observed on day 3 postinfection in mice infected with 100 plaque-forming units (PFU) of mutant virus compared to WT virus (Fig. 6D). Likewise, only mild weight loss (Fig. 6E) and complete survival were observed in mice infected with a dose of mutant virus, the equivalent of which was lethal using the WT parent strain.

### Efficacy of a single dose of MD185 or MD345 against a lethal influenza infection in mice

Mice were infected with a lethal dose of PR8 virus followed by intranasal treatment with a single 40-μg (2 mg/kg) dose of either compound at 48, 60, or 72 hpi (Fig. 7 and fig. S4). Zanamivir, which has comparable NA inhibiting function to the compounds (Fig. 2B), was used in parallel as a single treatment to highlight any enhancement of the compound’s activity attributable to the acidic domains. When treatment was initiated within 48 hpi, both MD185 and MD345 caused a significant reduction in morbidity and completely eliminated the need for euthanasia at the humane endpoint. Only when treatment was delayed to 60 hpi was a reduction in protection observed, with an 80% survival rate with both compounds. Even at 72 hpi, at a time point where the mice are already displaying clinical symptoms and substantial weight loss, MD185 and MD345 were able to rescue 60 and 70%, respectively, of the animals treated. This is in stark contrast to zanamivir, which showed no difference to the phosphate-buffered saline (PBS) control group, having no survivors. In humans, successful therapy at delayed time points is an important factor, given that many patients do not present until more than 48 hours after development of symptoms when currently licensed antivirals may prove much less effective (*20*).

**Fig. 7.**
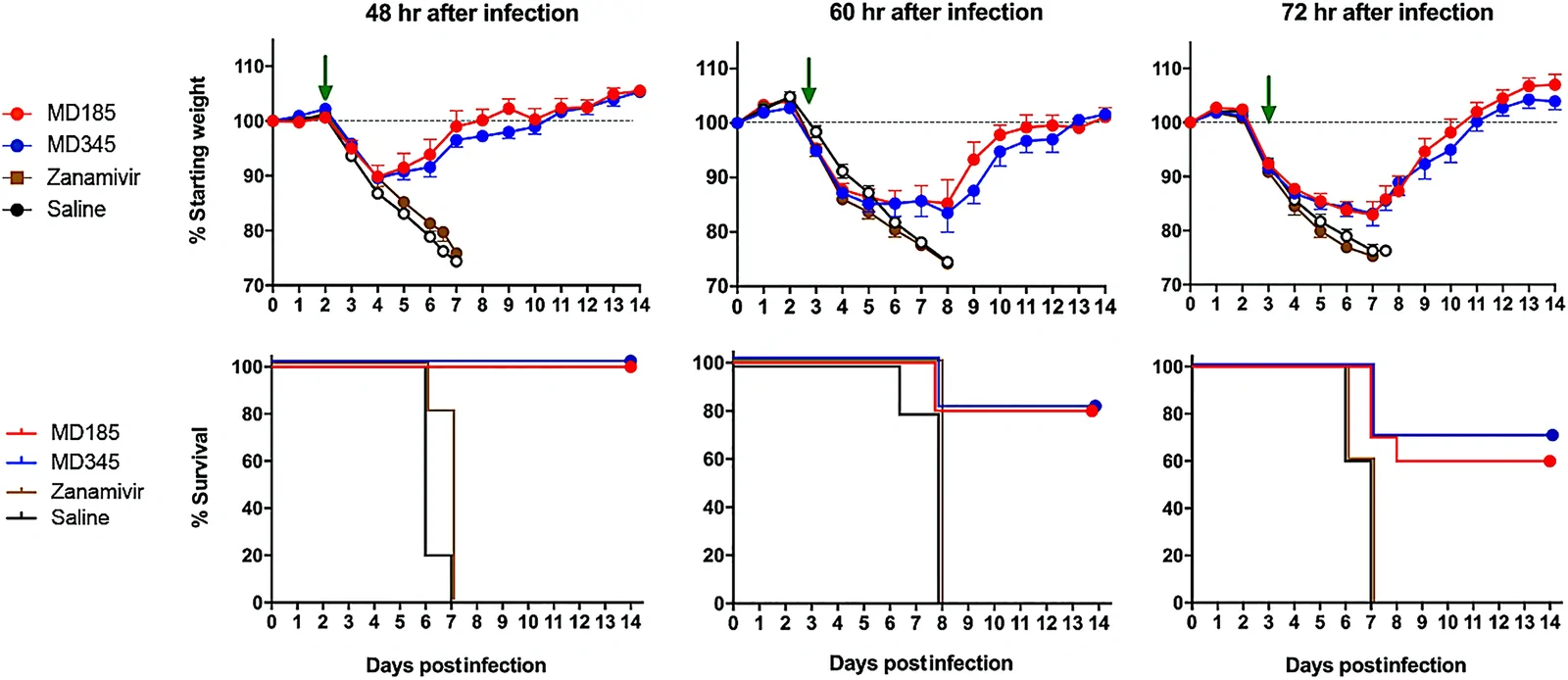
Therapeutic efficacy of the compounds in influenza infected mice. Mice were infected with a lethal dose (500 PFU) of PR8 virus and then treated intranasally with 40 μg (19.32 and 16.53 nmol, respectively) of MD185 (red), MD345 (blue), or zanamivir (brown) or saline (open black). Top: Means and SEM of weights of the mice in each group measured daily for 14 days after infection; the individual data for which is shown in fig. S4. Data are expressed as a percentage of the starting weights. Green arrows indicate time of treatment at 48, 60, or 72 hours after infection. Bottom: Survival curves of the same mice. Mantel-Cox tests of survival curves show no difference between the two compounds at any time point, and both compounds are significantly different from zanamivir (*P* = 0.0001 for administration at 48 hours, 0.015 at 60 hours, and 0.0037 at 72 hours). hr, hours.

To gain further insight into the action of the compounds, we determined the location and duration of activity of MD185 after i.n. delivery. Inhibitory activity was assessed in homogenized respiratory tissue from MD185-treated mice (Fig. 8) in a PRA against PR8 virus. Samples from 4 hours after delivery of 5 or 40 μg of MD185 inhibited PR8 virus from both nasal turbinates (NTs) and lung homogenates, with greater inhibition with 40 μg. Virus inhibitory activity declined over time but was still detectable after 7 days, the rate of decline being somewhat greater in the lungs than the NT. These data may suggest longevity of efficacy when the compounds are used in a prophylactic capacity.

**Fig. 8.**
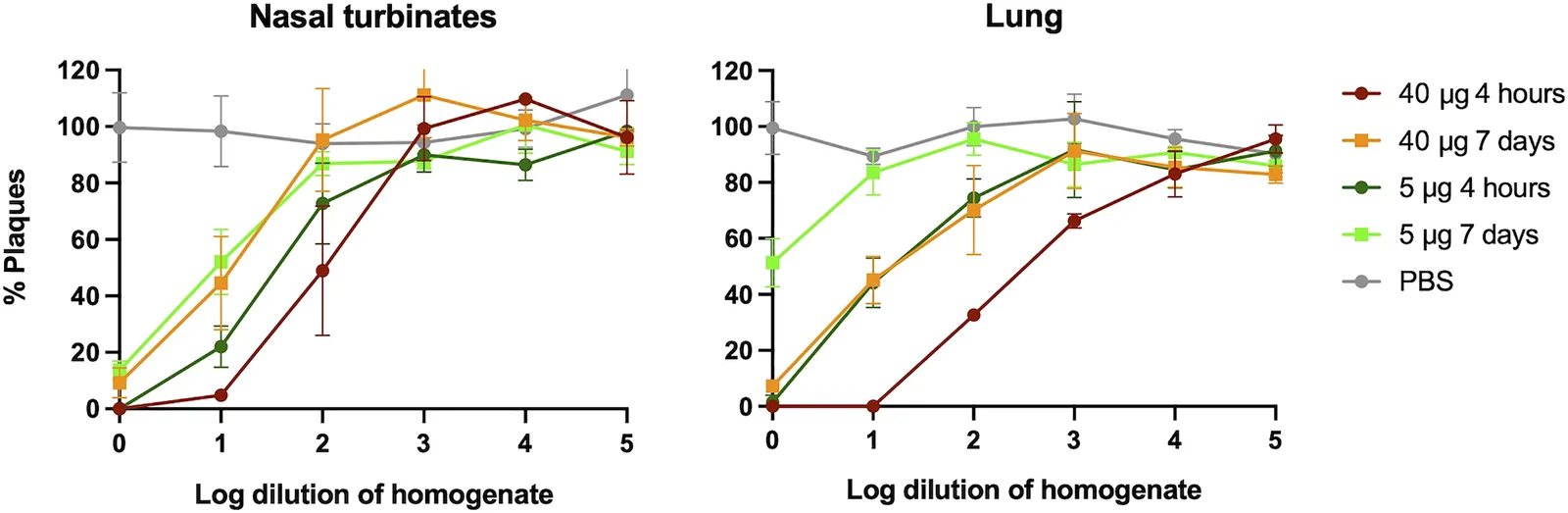
Measurement of compound activity in respiratory tissues. Mice were treated intranasally with either 5 μg (2.42 nmol) or 40 μg (19.32 nmol) of MD185 in 50 μl of PBS or 50 μl of PBS as control. At 4 hours or 7 days posttreatment, lungs and NTs were harvested and homogenized, and supernatants were treated with receptor-destroying enzyme (RDE). Virus inhibitory activity of the supernatants was assessed by PRA following incubation with PR8 virus with serial dilutions of extracts. Shown are the plaque numbers expressed as a percentage of a virus only assay control and represent the means and SEM of homogenates from three individual mice per group.

To test the prophylactic potential of the compounds, mice were given a single intranasal 5-μg (0.25 mg/kg) dose of either MD185, MD345, or zanamivir at 9, 11, or 13 days before infection with a lethal dose of PR8 (Fig. 9 and fig. S5). Mice survived when given MD185 or MD345 up to 11 days before infection. Although the MD185-treated group began to show weight loss when the time interval between administration of compound and infection was 11 days and mortality when the interval was increased to 13 days, the MD345-treated group showed no clinical signs at 13-day prophylactic interval, and, even then, all mice were protected from death. Notably, using only one fortieth of the dose, MD345 extended the duration of the protective effect beyond that observed with the most potent of zanamivir dimers (*21*). Overall, these data would indicate that, while the in vivo therapeutic and in vitro efficacy are similar between the two compounds, there appears to be a greater prophylactic capacity with MD345 compared with MD185, potentially due to differences in pharmacokinetic properties, such as rate of compound decay or removal from target sites, imparted by the different side chains.

**Fig. 9.**
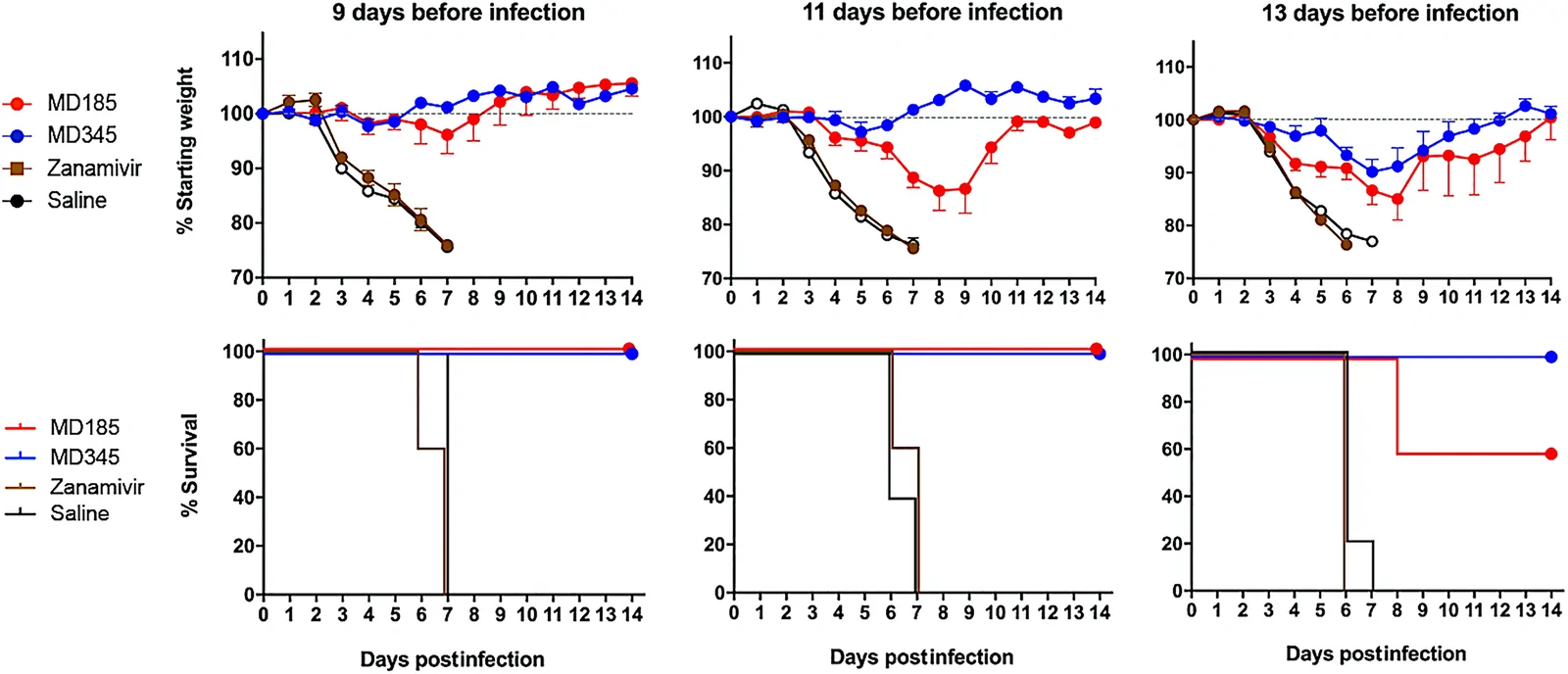
Prophylactic efficacy of the compounds in mice. Mice were given 5 μg (2.42 and 2.07 nmol, respectively) of MD185 (red), MD345 (blue), or zanamivir (brown) or saline (open black) intranasally either 9, 11, or 13 days before infection with a lethal dose (500 PFU) of PR8 virus. Data are presented as in Fig. 7. Weight change curves of MD185 and MD345 are significantly different only on day 11 (*P* = 0.0033, repeated measures ANOVA). Mantel-Cox tests of survival curves show no difference between the two compounds at any time point, and both compounds are significantly different from zanamivir (*P* = 0.0031 for administration at day −9, 0.0001 at day −11, and 0.0027 at day −13). Individual mouse data for this experiment are shown in fig. S5.

### Efficacy in ferrets

While the compounds were effective at preventing severe disease in mice, the murine model, which involves deposition of virus throughout the respiratory tract, does not accurately represent how influenza is acquired in nature. To explore the effectiveness of the compounds in a more representative model, outbred ferrets were given either 0.5 mg of MD185 intranasally either once only or once per day for 5 days, 0.5 mg of zanamivir intranasally once daily for 5 days, 5 mg of oseltamivir orally twice daily for 5 days, or PBS as a vehicle control, starting 2 hours before cohousing with A/Perth/265/09 (pdmH1N1) virus–infected donor ferrets (Fig. 10). All donor ferrets were confirmed to be shedding virus from day 1 postinfection (fig. S6A). Of the ferrets cohoused with the infected donors, three of the four in the placebo group had virus in the daily nasal washings detectable initially between 3 and 6 days after cohousing, and all had virus in at least one lung lobe when sampled on day 7 post-cohousing (Fig. 10 and fig. S6, B to D). Two of the four zanamivir-treated ferrets had virus in both nasal washings and lung lobes, while a third tested positive only in the lungs. Of the three ferrets treated twice daily with oseltamivir, one had virus in the day 5 and day 7 nasal washings, but none had virus in the lungs. In contrast, either a single treatment or five once-daily treatments of MD185 completely prevented the detection of virus in both the upper and lower respiratory tract. Notably, the single treatment with MD185 achieved comparable, if not better, protection than oseltamivir, even when the latter in a single day is given at a dose 20 times greater by weight and 119 times greater by molarity. Also of note, measurements of weight, temperature, and activity scores (fig. S7) showed no adverse indications in animals treated with a single dose or multiple doses of MD185.

**Fig. 10.**
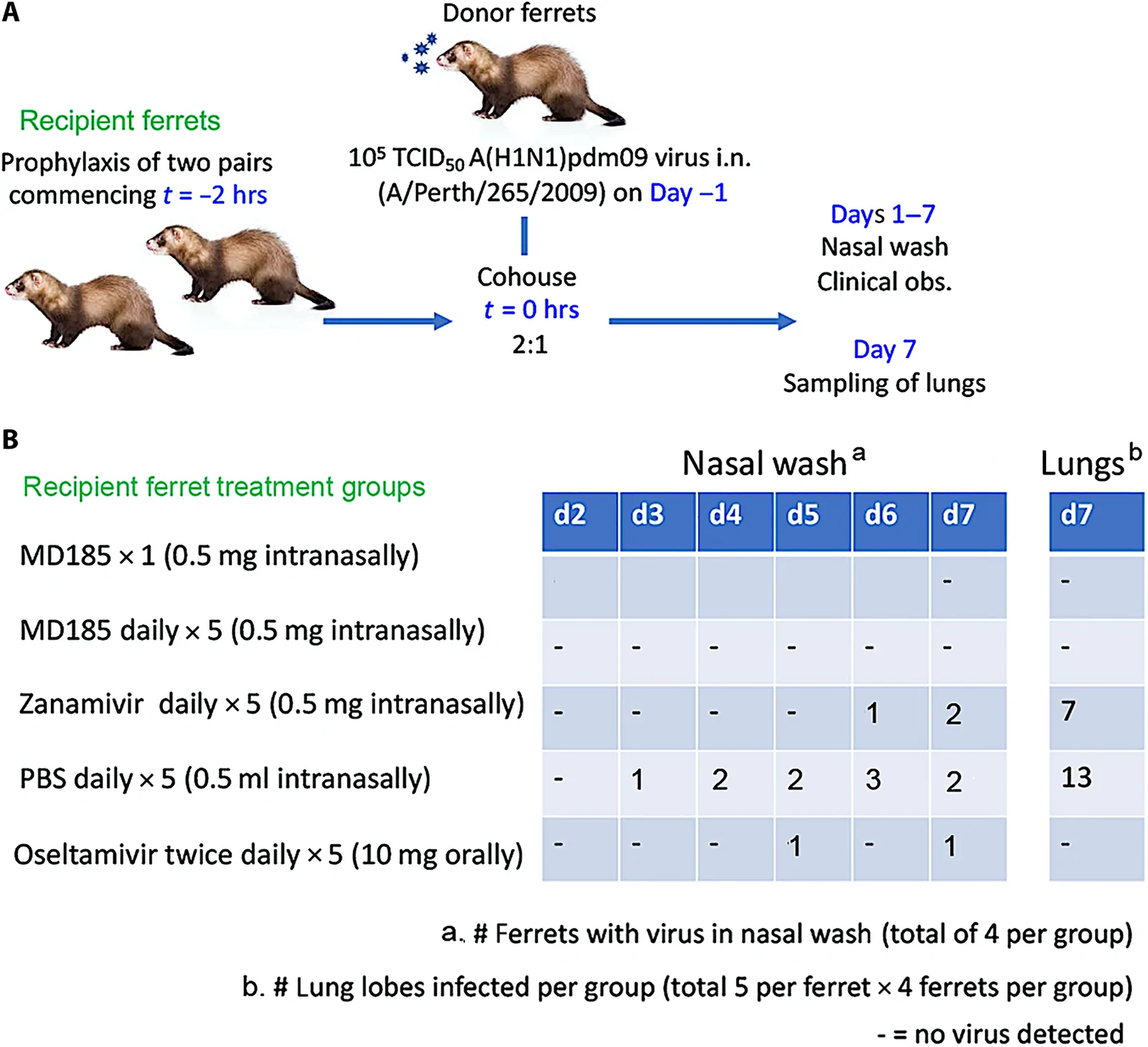
Ability of compounds to affect viral growth in the respiratory tract of ferrets. (**A**) Experimental design. Donor ferrets were infected and then cohoused with treated recipient ferrets as outlined. Nasal washings from the recipient ferrets were taken daily after exposure to the donor ferrets, except for the group treated with a single dose of MD185 that had nasal washings on day 7 only. Individual lung lobes of recipient ferrets were sampled on day 7 (ferret image from Adobe Stock). hrs, hours. (**B**) Summary of results for different treatment groups. Lungs and the nasal wash samples were assayed for infectious virus by a CPE assay in MDCK cells. For nasal washings, the number of virus-positive ferrets out of 4 is shown, - indicates no virus detected, and no symbol means not undergoing nasal washing. For the lungs, the data shown are the number of infected lobes out of a total of 20 per group. - indicates that no virus was detected. Titers for individual ferrets are shown in fig. S6. d, day.

## DISCUSSION

By targeting the acidic effector domain of the compounds to the virus surface, we were able to achieve prolonged and efficient inactivation of virus with a mode of action consistent with the creation of an acidic microenvironment at the virion surface that disabled virus entry into cells. Future imaging studies, such as cryo–electron microscopy of compound-treated virions may help confirm this mode of action. The highly successful application of our approach to influenza virus could provide the impetus to use this as a blueprint for the construction of a new class of antiviral compounds against the spectrum of viruses that use low pH as a trigger for endosomal membrane and for which premature acidification causes irreversible inhibition of infection [e.g., alphaviruses (*22*, *23*) and herpesviruses (*24*)]. Anchors such as nonneutralizing antibodies or their Fab fragments that bind in the vicinity of the fusion glycoprotein as well as other virus binding molecules such as integrin fragments could theoretically be engineered to target acidic domains to a viral surface. In the broader sense, this strategy might also be adapted for other pathogens for which inhospitable microenvironments and appropriate anchors can be envisaged.

To anchor our compounds to the virus, we used modified zanamivir. Although zanamivir has similar clinical efficacy to oseltamivir and both are administered twice daily for 5 days, zanamivir has found limited acceptance because it is taken via an inhaler device, compared with oseltamivir that is taken orally as a tablet. Yet zanamivir has two highly desirable properties, a high barrier to resistance and low toxicity, providing a valuable template for further development. Laninamivir (Inavir), a long-acting derivative of zanamivir, has been licensed in Japan where it has found better acceptance due to its single dosing (*25*). Other groups are also using zanamivir linked to conjugates including to an Fc (*26*), to haptens for which naturally occurring antibodies are abundant in humans (*27*), or to an anti-HA stem mAb (*28*). While showing promise, these also have limitations including ease of delivery or adverse effects (*29*). Our dual-acting compounds targeting both HA conformational integrity and NA function provide a major advantage, particularly against viruses that have reduced susceptibility to other antivirals.

The most recently approved influenza antiviral drug, baloxavir marboxil, a viral endonuclease inhibitor, also has a distinct advantage being administered as a single dose oral treatment. Although effective, the drug is prone to selection of resistant mutants (*30*), and these may be sufficiently fit to spread amongst individuals (*31*). Our in vivo data highlight the effectiveness of our compounds after a single small dose delivered intranasally, with no evidence of selection of fit mutants. Unlike zanamivir, the long prophylactic window seen particularly with MD345 suggests a potential role for postexposure prophylaxis and for prevention, both highly desirable in a pandemic context.

## MATERIALS AND METHODS

### Viruses and cells

MDCK cells were grown in RPMI 1640 medium (Sigma-Aldrich, Castle Hill, Australia) supplemented with 10% heat-inactivated fetal calf serum (FCS; Life Technologies, Mulgrave, Australia), 2 mM l-glutamine (Sigma-Aldrich), 2 mM sodium pyruvate, gentamicin (24 μg/ml; Pfizer, West Ryde, NSW, Australia), streptomycin (50 μg/ml), and penicillin (50 IU/ml), unless otherwise stated. MDCK-SIAT 1 cells were obtained from the WHO Influenza Centre for Reference and Research, Melbourne, and were grown in the same medium with additional Geneticin/G418 (1 mg/ml) to maintain the alpha-2,6 sialyl transferase gene. Influenza viruses—A/Puerto Rico/8/34 (H1N1), A/Memphis/1/71 (H3N2), A/California/7/09 (pdmH1N1), A/Switzerland/9715293/2013 (H3N2), and B/Malaysia/2008—were from the collection at the Peter Doherty Institute and were amplified in 10-day embryonated hen’s eggs. Stocks of A/Perth/265/09 (pdmH1N1), A/Fukui/45/04 (H3N2), A/ Mississippi/3/2001 (H1N1) and its H274Y oseltamivir-resistant mutant, and A/NWS/G7OC (H1N9) and its E119G zanamivir-resistant mutant were obtained from CSIRO Materials Science and Engineering, Parkville, and amplified in MDCK cells.

### Viral CPE-reduction assay

This assay was used for the initial screening of compounds against a range of virus strains. MDCK cells were maintained in minimal essential medium (Gibco, Thermo Fisher Scientific) supplemented with 1 mM nonessential amino acids, penicillin (50 U/ml), streptomycin (50 μg/ml), 1 mM sodium pyruvate, and 10% fetal bovine serum. For assay of compounds, MDCK cells were grown to confluency in 96-well microtiter trays. Medium was removed, and 46 μl of virus at 10-fold above the 50% tissue culture infectious dose (TCID_50_) was added to each well. Plates were incubated at 37°C for 1.5 hours after which the viral inoculum was removed and replaced with medium containing trypsin [l-1-tosylamido-2-phenylethyl chloromethyl ketone (TPCK) treated]. Test compounds were serially diluted 1:3 in the wells. Control wells contained medium in place of compound. After 48 to 96 hours, the medium was removed and replaced with medium without trypsin. Viable cells were detected after the addition of CellTiter 96 Aqueous One Solution reagent (Promega) by measurement of absorbance at 490 nm, according to the manufacturer’s instructions.

### Cellular toxicity assay

Fifty percent confluent MDCK cells in a 96-well plate were treated with half-log dilutions in RPMI, supplemented with 10% FCS, penicillin (50 U/ml), streptomycin (50 μg/ml), and GlutaMAX (Gibco), of either MD185 or MD345, starting at 100 μM per well, or with PBS. At 68 hours posttreatment, additional wells were treated with half-log dilutions of Triton X-100 (Sigma-Aldrich) starting at 0.01% v/v in the above medium as a positive control. At 72 hours posttreatment, cell toxicity was analyzed using the CellTiter-Glo Luminescent Cell Viability Assay (Promega) according to the kit instructions: The culture plate was equilibrated to room temperature, 100 μl of CellTiter-Glo reagent was added to each well, and the plate was left on an orbital shaker for 12 min at room temperature. One hundred microliters was transferred from each well to a white opaque 96-well plate and read on a FLUOstar Omega (BMG Labtech).

### Assay of infectious virus by plaque formation

Viruses were enumerated by plaque assay performed in duplicate wells in TC-6 plates (Corning) of confluent MDCK cell monolayers overlaid with 3 ml of Leibovitz’s L-15 medium with l-glutamine (Gibco), 0.2 mM Hepes buffer, 0.9% (w/v) type 1 agarose (Sigma-Aldrich), and TPCK-treated trypsin (1 μg/ml; Worthington Biochemicals). After 3 days of culture, PFUs were counted either directly or after fixing overnight in 1% formaldehyde in saline and staining with 0.05% neutral red.

### Plaque reduction assays

MD185 and MD345 were dissolved in RPMI at a concentration of 200 nM, and zanamivir was reconstituted to 2000 nM before each sample was 10-fold serially diluted to 0.001 nM. Influenza virus at 1 × 10^3^ PFU/ml in RPMI was mixed 1:1 with compound dilutions and preincubated at room temperature for 1 hour. Confluent MDCK monolayers in six-well plates were washed with RPMI, and 200 μl per well of the virus/compound dilutions were added to triplicate wells. The plates were left at 37°C for 1 hour before being overlaid with medium containing agarose as above. As the distinguishing mode of action of the compounds compared with zanamivir is to inhibit virus entry in addition to inhibiting the NA, compounds were preincubated with virus, similar to a neutralization assay, and, at 3 days postinfection, the numbers of plaques readily visible after neutral red staining were counted.

### NA assay with fetuin substrate

A 96-well Maxisorp plate (Thermo Fisher Scientific, Denmark) was coated with 100 μl of fetuin (125 μg/ml; Sigma-Aldrich) in PBS and left overnight at 4°C. Virus was serially diluted to 5 × 10^7^ PFU/ml in stability buffer (0.9% NaCl, 1% bovine serum albumen, 10 mM CaCl_2_, and 5 mM MgCl_2_) containing 0.5% Triton X-100 to separate the activity of the NA from the binding capacity of the HA and left at room temperature for 20 min. The fetuin plate was washed three times with PBS, and each dilution (100 μl per well) was added in duplicate before the plate was incubated at 37°C for 16 hours. The plate was washed six times in PBS with 0.05% Tween 20 (PBST) and then incubated with horseradish peroxidase (HRP)–conjugated peanut *Arachis hypogaea* lectin (Cosmo Bio) for 1 hour at room temperature. Further washes in PBST were performed before SureBlue TMB substrate (100 μl per well; SeraCare) was added and left at room temperature for 20 min. The reaction was stopped with 1 M HCl (50 μl per well) and read at 450 nm. For NA inhibition assays, MD185, MD345, or zanamivir (45 μM) was 10-fold serially diluted in stability buffer and then incubated 1:1 with virus (5 × 10^7^ PFU/ml) for 20 min at room temperature before adding to the washed fetuin plate.

### NA inhibition assay with MUNANA substrate

NA activity was measured using the MUNANA fluorescence–based assay, as described previously (*32*). The assay measures the increase in fluorescence of free methylumbelliferone released from MUNANA after cleavage by NA. Viruses, diluted to generate comparable fluorescence units, were preincubated in microtiter plates for 30 min at 37°C with serial twofold dilutions of compound. MUNANA (Carbosynth) was then added, the assay mixtures were incubated for 1 hour at 37°C, and the assays were stopped by the addition of 0.2 M Na_2_CO_3_. Fluorescence was measured in a BMG FLUOstar Optima reader with an excitation wavelength of 365 nm and an emission wavelength of 450 nm.

### Slot blot assay

A/Mem/l/71-Bel/42 (H3N1) (3.4 × 10^7^ PFU/ml) was diluted 1 in 10 in either pH 7.2 saline, pH 4.9 10 mM sodium citrate buffer, or 340 μM MD345 or MD185 in pH 7.2 saline and left at room temperature for 2 hours. Each virus sample was then twofold serially diluted in saline before 50 μl was added to triplicate wells of a Minifold 2 slot blot apparatus (*33*). After binding, filters were removed, cut into three replicate strips, and blocked with 4 ml of 1% casein in PBS for 10 min. Separate strips were then reacted for 1 hour with one of the three mAbs: mAb 88-2 that detects only the low-pH conformation of HA, mAb 241 that detects only the neutral-pH conformation, or mAb 244 that can bind to both conformations. After washing, an HRP-conjugated rabbit anti-mouse antibody (Dako) was applied for 1 hour, strips were washed, and SureBlue TMB substrate (SeraCare) with 1% dextran sulfate was added to each strip. After developing, the strips were rinsed with water, dried, and scanned with a DocuPrint CM415 AP Fuji xerox scanner. Band density was analyzed using ImageJ software analogous to methods used for quantifying bands in gels.

### Hemagglutination inhibition

PR8 virus (2.6 × 10^4^ PFU) was preincubated in Eppendorf tubes with either saline or the indicated amounts of MD185, MD345 or zanamivir. After 2 hours, serial twofold dilutions of the mixtures were performed in round-bottomed macro well plates, and an equivalent volume of 1% (v/v) chicken erythrocytes was added. The pattern of hemagglutination was photographed after 30 min.

### Time of addition assay

Monolayers of MDCK cells in 96-well plates were infected with an MOI of 0.1 PFU per cell starting at *t* = −1 hour. Duplicate wells were treated with 5 μg of either MD185 or zanamivir in 100 μl of RPMI 1640 medium. Each treatment occurred for a period of 2 hours: pretreatment of cells with compound in the absence of virus, from −4 to −2 hours; attachment and entry, a 1-hour preincubation of virus and compound from −2 to −1 hour followed by addition to cell monolayer from −1 to 0 hours; early replication, from 0 to 2 hours; late replication, from 2 to 4 hours; and packaging and exit, from 4 to 6 hpi. Control wells were left untreated. Following treatment, cells were washed extensively to remove any trace of compound; this involved two initial washes followed by two 30-min washes in 200 μl of RPMI. Supernatants were harvested at 12 hpi to maximize virus yields from the first cycle but minimize potential release of virus from the second round of replication. Samples were serially diluted, and virus was assayed by plaque formation in MDCK monolayers.

### Selection and sequencing of resistant mutants

Tenfold serial dilutions of A/Perth/265/09 (pdmH1N1) and A/Fukui/45/04 (H3N2) viruses were passaged at a multiplicity of >1.0 in MDCK cells in 24-well dishes for four passages. MD185 was added at 10 ng/ml, and control wells included virus without any inhibitor to detect any adaptive mutations. Virus titer was determined after each passage by plaque assay, and plaque morphology was examined for phenotypic changes indicative of potential mutations, which were seen by passage 4. Potential mutants were plaque purified in MDCK monolayers and picked into 200 μl of RPMI 1640 medium. Viral amplification was performed in chicken egg embryos for 48 hours at 35°C or MDCK cells for 72 hours at 37°C. RNA was extracted from viral supernatants using the RNeasy mini kit (QIAGEN, Germany) followed by cDNA conversion using the Omniscript RT kit (QIAGEN, Germany) according to kit instructions. DNA was amplified using a Phusion Flash PCR master mix (Thermo Fisher Scientific) with HA- and NA- specific primers (primers available on request). The samples were gel purified using the QIAquick gel extraction kit (QIAGEN, Germany), and sequencing was performed using the BigDye Terminator v3.1 Cycle Sequencing Kit (Applied Biosystems, Massachusetts, USA).

### Mouse infection experiments

Experiments were conducted under approval from the University of Melbourne’s Animal Ethics Committee (projects 1112112.3, 1212572.4, 1212665.4, 1413321.3, and 1714217.2) in accordance with the Australian code for the care and use of animals for scientific purposes. Seven- to 8-week-old female BALB/c were obtained from the Animal Resources Centre (Perth, Australia) and housed at the Bioresources Facility at the Peter Doherty Institute. The mice were anesthetized using isoflurane and treated intranasally with 5 μg per mouse for prophylaxis or 40 μg per mouse for therapy of MD185, MD345, or zanamivir in 50 μl of 0.9% saline. At the specified times pre- or posttreatment, the animals were anesthetized again and infected intranasally with a lethal dose (500 PFU) of PR8 in 50 μl of 0.9% saline. All infected animals were weighed and monitored for clinical signs at least once daily and euthanized at the predefined humane endpoint that consists of (i) weight loss of 20% or more detected over a 2-day period and accompanied by drop in body temperature and abnormal breathing and heart rate or (ii) where condition has led to inability to eat or drink, e.g., severe hunching and inactivity.

In some experiments, C57BL/6 mice (Peter Doherty Institute) were infected with variants of A/Perth/265/09 virus selected in the presence of MD185 or with the WT parent virus. Lungs were harvested from the mice on day 3 after infection and assayed for infectious virus by plaque formation in MDCK cells. Ethics approval for these experiments was granted after dose escalation studies in BALB/c mice with doses up to 200 μg per mouse, which showed no adverse effects with either compound.

### Preparation of mouse tissue homogenates

C57BL/6 mice were treated intranasally under anesthetic with MD185 in 50 μl of PBS or with 50 μl of PBS as control. At 4 hours or 7 days posttreatment, mice were euthanized with CO_2_, and lungs and NTs were harvested into 2 ml of serum-free RPMI (+pen/strep and GlutaMAX). Tissues were homogenized and spun at 1000*g* for 5 min, and supernatants were collected. Supernatants were diluted 1:1 with receptor-destroying enzyme, RDE (II) (Denka-Seiken), to remove innate inhibitors of the virus, and left overnight at 37°C. Samples were then heated to 56°C for 30 min to inactivate the RDE and additional virus inhibitors, before being serially diluted in serum-free RPMI and mixed 1:1 with PR8 virus (1 × 10^3^ PFU/ml). Samples were left at room temperature for 60 min before 200 μl was assayed by plaque formation on confluent MDCK cells in six-well plates, as described above.

### Ferret infection experiments

Experiments were conducted under approval from the University of Melbourne’s Animal Ethics Committee (project 1313040) in accordance with the Australian code for the care and use of animals for scientific purposes. Six- to 12-month-old outbred ferrets (12 females and 17 males *Mustela putorius furo*; Animalactic Animals and Animal Products, Australia), with weights ranging from 891 to 1316 g, were housed at the Bioresources Facility within the Peter Doherty Institute. The day before cohousing, 10 female donor ferrets were anesthetized using an intramuscular xylazine:ketamine injection before being infected intranasally with 10^5^ TCID_50_ A/Perth/265/2009 virus. The remaining animals were randomly allocated into one of the five treatment groups: 0.5 mg of MD185 given intranasally once only; 0.5 mg of MD185 given intranasally once a day for 5 days; 0.5 mg of zanamivir given intranasally once a day for 5 days; PBS placebo given once a day for 5 days; or 5 mg of oseltamivir phosphate (Sequoia Research Products) given orally twice a day for 5 days, all in 500-μl volumes. All treatments were performed under ketamine:midazolam:medetomidine anesthesia except for the oseltamivir group where drug was administered to conscious ferrets. Two hours after the initial treatment, one donor ferret was cohoused with two recipient ferrets for 48 hours before the donor was removed. The recipient animals were weighed daily, and changes in body temperature were assessed using implanted temperature transponders fitted to identification chips (LifeChip Bio-Thermo, Digivet, Australia). Ferret activity was assessed daily at the same time by placing a single ferret into a plastic box and recording movement on a video camera for 1 to 5 min after 7 s of acclimatization (*34*). Activity (pixel change) was measured using an automated video-tracking and motion analysis program, EthoVision XT 10.0 (Noldus IT, The Netherlands). Weight and activity score data are presented as a percentage of the baseline score for each ferret. Nasal washes were taken starting 24 hpi to determine viral titers. A nasal wash was performed on the MD185 single administration group only once on day 7 due to fears that residual compound would be washed away. Seven days after cohousing, all animals were euthanized and individual lung lobes dissected for analysis of virus titers using a CPE assay. Titers of virus in nasal washes and lung homogenates were expressed as TCID_50_/ml determined in MDCK cells.

### Statistical analysis

All statistical analyses were performed with GraphPad Prism version 9.4.1 using one-way or two-way analysis of variance (ANOVA) as indicated, using Šidák’s multiple comparisons tests unless otherwise specified.

## References

[1] J. L. McKimm-Breschkin, Influenza neuraminidase inhibitors: Antiviral action and mechanisms of resistance. Influenza Other Respi. Viruses 7, 25–36 (2013). 10.1111/irv.12047 PMC4942987 23279894

[2] E. J. Mifsud, F. G. Hayden, A. C. Hurt, Antivirals targeting the polymerase complex of influenza viruses. Antiviral Res. 169, 104545 (2019). 10.1016/j.antiviral.2019.104545 31247246

[3] W. Weis, J. H. Brown, S. Cusack, J. C. Paulson, J. J. Skehel, D. C. Wiley, Structure of the influenza virus haemagglutinin complexed with its receptor, sialic acid. Nature 333, 426–431 (1988). 10.1038/333426a0 3374584

[4] P. A. Bullough, F. M. Hughson, J. J. Skehel, D. C. Wiley, Structure of influenza haemagglutinin at the pH of membrane fusion. Nature 371, 37–43 (1994). 10.1038/371037a0 8072525

[5] J. J. Skehel, D. C. Wiley, Receptor binding and membrane fusion in virus entry: The influenza hemagglutinin. Annu. Rev. Biochem. 69, 531–569 (2000). 10.1146/annurev.biochem.69.1.531 10966468

[6] S. C. Harrison, Viral membrane fusion. Nat. Struct. Mol. Biol. 15, 690–698 (2008). 10.1038/nsmb.1456 PMC2517140 18596815

[7] C. M. Carr, C. Chaudhry, P. S. Kim, Influenza hemagglutinin is spring-loaded by a metastable native conformation. Proc. Natl. Acad. Sci. U.S.A. 94, 14306–14313 (1997). 10.1073/pnas.94.26.14306 PMC24954 9405608

[8] S. B. Sato, K. Kawasaki, S. Ohnishi, Hemolytic activity of influenza virus hemagglutinin glycoproteins activated in mildly acidic environments. Proc. Natl. Acad. Sci. U.S.A. 80, 3153–3157 (1983). 10.1073/pnas.80.11.3153 PMC393998 6574476

[9] M. Linster, S. van Boheemen, M. de Graaf, E. J. A. Schrauwen, P. Lexmond, B. Manz, T. M. Bestebroer, J. Baumann, D. van Riel, G. F. Rimmelzwaan, A. Osterhaus, M. Matrosovich, R. A. M. Fouchier, S. Herfst, Identification, characterization, and natural selection of mutations driving airborne transmission of A/H5N1 virus. Cell 157, 329–339 (2014). 10.1016/j.cell.2014.02.040 PMC4003409 24725402

[10] M. von Itzstein, W. Y. Wu, G. B. Kok, M. S. Pegg, J. C. Dyason, B. Jin, T. Van Phan, M. L. Smythe, H. F. White, S. W. Oliver, P. M. Colman, J. N. Varghese, D. M. Ryan, J. M. Woods, R. C. Bethell, V. J. Hotham, J. M. Cameron, C. R. Penn, Rational design of potent sialidase-based inhibitors of influenza virus replication. Nature 363, 418–423 (1993). 10.1038/363418a0 8502295

[11] J. N. Varghese, P. W. Smith, S. L. Sollis, T. J. Blick, A. Sahasrabudhe, J. L. McKimm-Breschkin, P. M. Colman, Drug design against a shifting target: A structural basis for resistance to inhibitors in a variant of influenza virus neuraminidase. Structure 6, 735–746 (1998). 10.1016/s0969-2126(98)00075-6 9655825

[12] S. J. Macdonald, K. G. Watson, R. Cameron, D. K. Chalmers, D. A. Demaine, R. J. Fenton, D. Gower, J. N. Hamblin, S. Hamilton, G. J. Hart, G. G. Inglis, B. Jin, H. T. Jones, D. B. McConnell, A. M. Mason, V. Nguyen, I. J. Owens, N. Parry, P. A. Reece, S. E. Shanahan, D. Smith, W. Y. Wu, S. P. Tucker, Potent and long-acting dimeric inhibitors of influenza virus neuraminidase are effective at a once-weekly dosing regimen. Antimicrob. Agents Chemother. 48, 4542–4549 (2004). 10.1128/AAC.48.12.4542-4549.2004 PMC529190 15561823

[13] R. G. Webster, L. E. Brown, D. C. Jackson, Changes in the antigenicity of the hemagglutinin molecule of H3 influenza virus at acidic pH. Virology 126, 587–599 (1983). 10.1016/s0042-6822(83)80015-4 6190310

[14] L. E. Brown, J. M. Murray, D. O. White, D. C. Jackson, An analysis of the properties of monoclonal antibodies directed to epitopes on influenza virus hemagglutinin. Arch. Virol. 114, 1–26 (1990). 10.1007/BF01311008 1699509

[15] T. J. Blick, T. Tiong, A. Sahasrabudhe, J. N. Varghese, P. M. Colman, G. J. Hart, R. C. Bethell, J. L. McKimm-Breschkin, Generation and characterization of an influenza virus neuraminidase variant with decreased sensitivity to the neuraminidase-specific inhibitor 4-guanidino-Neu5Ac2en. Virology 214, 475–484 (1995). 10.1006/viro.1995.0058 8553549

[16] A. S. Monto, J. L. McKimm-Breschkin, C. Macken, A. W. Hampson, A. Hay, A. Klimov, M. Tashiro, R. G. Webster, M. Aymard, F. G. Hayden, M. Zambon, Detection of influenza viruses resistant to neuraminidase inhibitors in global surveillance during the first 3 years of their use. Antimicrob. Agents Chemother. 50, 2395–2402 (2006). 10.1128/AAC.01339-05 PMC1489772 16801417

[17] K. Little, S.-K. Leang, J. Butler, C. Baas, B. Harrower, J. Mosse, I. G. Barr, A. C. Hurt, Zanamivir-resistant influenza viruses with Q136K or Q136R neuraminidase residue mutations can arise during MDCK cell culture creating challenges for antiviral susceptibility monitoring. Euro Surveill. 20, 10.2807/1560-7917.ES.2015.20.45.30060 (2015). 26608955

[18] J. L. McKimm-Breschkin, S. Barrett, C. McKenzie-Kludas, J. McAuley, V. A. Streltsov, S. G. Withers, Passaging of an influenza A(H1N1)pdm09 virus in a difluoro sialic acid inhibitor selects for a novel, but unfit I106M neuraminidase mutant. Antiviral Res. 169, 104542 (2019). 10.1016/j.antiviral.2019.104542 31233807

[19] Y. Abed, X. Bouhy, A. G. L’Huillier, C. Rheaume, A. Pizzorno, M. Retamal, C. Fage, K. Dube, M. H. Joly, E. Beaulieu, C. Mallett, L. Kaiser, G. Boivin, The E119D neuraminidase mutation identified in a multidrug-resistant influenza A(H1N1)pdm09 isolate severely alters viral fitness in vitro and in animal models. Antiviral Res. 132, 6–12 (2016). 10.1016/j.antiviral.2016.05.006 27185624

[20] T. M. Uyeki, H. H. Bernstein, J. S. Bradley, J. A. Englund, T. M. File, A. M. Fry, S. Gravenstein, F. G. Hayden, S. A. Harper, J. M. Hirshon, M. G. Ison, B. L. Johnston, S. L. Knight, A. McGeer, L. E. Riley, C. R. Wolfe, P. E. Alexander, A. T. Pavia, Clinical practice guidelines by the Infectious Diseases Society of America: 2018 Update on diagnosis, treatment, chemoprophylaxis, and institutional outbreak management of seasonal influenza. Clin. Infect. Dis. 68, 895–902 (2019). 10.1093/cid/ciy874 PMC6769232 30834445

[21] E. B. Tarbet, S. Hamilton, A. H. Vollmer, A. Luttick, W. C. Ng, M. Pryor, B. L. Hurst, S. Crawford, D. F. Smee, S. P. Tucker, A zanamivir dimer with prophylactic and enhanced therapeutic activity against influenza viruses. J. Antimicrob. Chemother. 69, 2164–2174 (2014). 10.1093/jac/dku127 PMC4100710 24777908

[22] C. Sanchez-San Martin, C. Y. Liu, M. Kielian, Dealing with low pH: Entry and exit of alphaviruses and flaviviruses. Trends Microbiol. 17, 514–521 (2009). 10.1016/j.tim.2009.08.002 PMC2783195 19796949

[23] J. Edwards, E. Mann, D. T. Brown, Conformational changes in Sindbis virus envelope proteins accompanying exposure to low pH. J. Virol. 45, 1090–1097 (1983). 10.1128/jvi.45.3.1090-1097.1983 PMC256517 6834477

[24] D. J. Weed, S. M. Pritchard, F. Gonzalez, H. C. Aguilar, A. V. Nicola, Mildly acidic pH triggers an irreversible conformational change in the fusion domain of herpes simplex virus 1 glycoprotein B and inactivation of viral entry. J. Virol. 91, e02123–e02216 (2017). 10.1128/JVI.02123-16 PMC5309949 28003487

[25] S. Sunagawa, F. Higa, H. L. Cash, M. Tateyama, T. Uno, J. Fujita, Single-dose inhaled laninamivir: Registered in Japan and its potential role in control of influenza epidemics. Influenza Other Respi. Viruses 7, 1–3 (2013). 10.1111/j.1750-2659.2012.00351.x PMC5780724 22405585

[26] S. Dohrmann, J. Levin, J. N. Cole, A. Borchardt, K. Amundson, A. Almaguer, E. Abelovski, R. Grewal, D. Zuill, N. Dedeic, G. Hough, J. Fortier, J. Donatelli, T. Lam, Z. Y. Chen, W. Jiang, T. Haussener, A. Noncovich, J. M. Balkovec, D. C. Bensen, V. Ong, T. P. Brady, J. B. Locke, S. Flanagan, R. M. Hughes, J. L. Stein, L. W. Tari, Drug-Fc conjugate CD388 targets influenza virus neuraminidase and is broadly protective in mice. Nat. Microbiol. 10, 912–926 (2025). 10.1038/s41564-025-01955-3 PMC11964917 40097766

[27] I. Shahriar, M. Kamra, A. K. Kanduluru, C. L. Campbell, T. H. Nguyen, M. Srinivasarao, P. S. Low, Targeted recruitment of immune effector cells for rapid eradication of influenza virus infections. Proc. Natl. Acad. Sci. U.S.A. 121, e2408469121 (2024). 10.1073/pnas.2408469121 PMC11474073 39348541

[28] X. Liu, T. Balligand, C. Le Gall, H. L. Ploegh, A monoclonal anti-hemagglutinin stem antibody modified with zanamivir protects against both influenza A and B viruses. Proc. Natl. Acad. Sci. U.S.A. 122, e2424889122 (2025). 10.1073/pnas.2424889122 PMC12012527 40193611

[29] S. O. Ali, T. Takas, A. Nyborg, K. Shoemaker, N. L. Kallewaard, R. Chiong, F. Dubovsky, R. M. Mallory, Evaluation of MEDI8852, an anti-influenza A monoclonal antibody, in treating acute uncomplicated influenza. Antimicrob. Agents Chemother. 62, e00694–e00718 (2018). 10.1128/AAC.00694-18 PMC6201130 30150460

[30] T. Uehara, F. G. Hayden, K. Kawaguchi, S. Omoto, A. C. Hurt, M. D. De Jong, N. Hirotsu, N. Sugaya, N. Lee, K. Baba, T. Shishido, K. Tsuchiya, S. Portsmouth, H. Kida, Treatment-emergent influenza variant viruses with reduced baloxavir susceptibility: Impact on clinical and virologic outcomes in uncomplicated ifluenza. J. Infect. Dis. 221, 346–355 (2020). 10.1093/infdis/jiz244 31309975

[31] E. Takashita, M. Ichikawa, H. Morita, R. Ogawa, S. Fujisaki, M. Shirakura, H. Miura, K. Nakamura, N. Kishida, T. Kuwahara, H. Sugawara, A. Sato, M. Akimoto, K. Mitamura, T. Abe, M. Yamazaki, S. Watanabe, H. Hasegawa, T. Odagiri, Human-to-human transmission of influenza A(H3N2) virus with reduced susceptibility to baloxavir, Japan, February 2019. Emerg. Infect. Dis. 25, 2108–2111 (2019). 10.3201/eid2511.190757 PMC6810216 31436527

[32] J. L. McKimm-Breschkin, C. Rootes, P. G. Mohr, S. Barrett, V. A. Streltsov, In vitro passaging of a pandemic H1N1/09 virus selects for viruses with neuraminidase mutations conferring high-level resistance to oseltamivir and peramivir, but not to zanamivir. J. Antimicrob. Chemother. 67, 1874–1883 (2012). 10.1093/jac/dks150 22563014

[33] J. L. McKimm-Breschkin, The use of tetramethylbenzidine for solid phase immunoassays. J. Immunol. Methods 135, 277–280 (1990). 10.1016/0022-1759(90)90282-z 2273262

[34] D. Y. Oh, I. G. Barr, A. C. Hurt, A novel video tracking method to evaluate the effect of influenza infection and antiviral treatment on ferret activity. PLOS ONE 10, e0118780 (2015). 10.1371/journal.pone.0118780 PMC4349809 25738900

